# Social behaviour in mesopelagic jellyfish

**DOI:** 10.1038/srep11310

**Published:** 2015-06-11

**Authors:** Stein Kaartvedt, Karl I. Ugland, Thor A. Klevjer, Anders Røstad, Josefin Titelman, Ingrid Solberg

**Affiliations:** 1 University of Oslo, Department of Biosciences, PO Box 1066 Blindern, 0316 Oslo, Norway; 2 King Abdullah University of Science and Technology, Red Sea Research Center, Thuwal 23955-6900, Saudi Arabia; 3 Institute of Marine research, POBox 1870 Nordnes, 5817 Bergen, Norway

## Abstract

Gelatinous organisms apparently play a central role in deep pelagic ecosystems, but lack of observational methodologies has restricted information on their behaviour. We made acoustic records of diel migrating jellyfish *Periphylla periphylla* forming small, ephemeral groups at the upper fringe of an acoustic scattering layer consisting of krill. Groups of *P. periphylla* were also documented photographically using a remotely operated vehicle (ROV). Although the adaptive value of group formation remains speculative, we clearly demonstrate the ability of these jellyfishes to locate and team up with each other.

Deep pelagic habitats are characterized by dark waters, dilute populations, few conspecifics and scarce food, and animals evidently require unique physiological and behavioural adaptations to succeed in this realm^1^. From the use of manned submersibles and remotely operated vehicles (ROVs) it has become increasingly apparent that gelatinous forms play a central role in deep pelagic ecosystems^1^,^2^. Yet, with the low concentrations of organisms present only scattered observations exist and lack of observational methodologies generally restricts behavioural information from this inaccessible part of the ocean.

In Lurefjorden, Norway, the cosmopolitan mesopelagic jellyfish *Periphylla periphylla* occurs in concentrations several orders of magnitude higher and at shallower depths than in its normal oceanic habitat^3^,^4^,^5^, offering unmatched opportunities to study behavioural processes, that are otherwise difficult to observe in deep water jellyfish. The high abundance in Lurefjorden has been ascribed to the particularly high light extinction of the water in this basin, which may have excluded visually foraging mesopelagic fish from the dark, deep waters^6^,^7^. This in turn leaves a surplus of prey for the jellyfish^6^,^8^.

The use of echo sounders enables the observation of individual jellyfish *in situ*, and acoustic studies have documented strikingly diverse behavioural patterns among *P. periphylla*, with the population segregating into assemblages with distinctly different vertical distribution and diel vertical migration (DVM) behaviour^5^. Individuals constituting the shallow-most of these assemblages (behavioural Mode 1, *sensu* 5) inhabit the upper 100 m, where they carry out synchronous DVM. These are the jellyfish addressed in this study. Nocturnal groups of *P. periphylla* have previously been reported at the surface and ascribed to reproduction, as seeking the two dimensional surface would increase the chance of encounter and mating compared to the three dimensional water column^9^. We here show that *P. Periphylla* readily teams up also at depth, forming ephemeral groups of a few individuals that may stay together for 10’s of minutes.

## Results

### Acoustic studies

**Figure 1:**
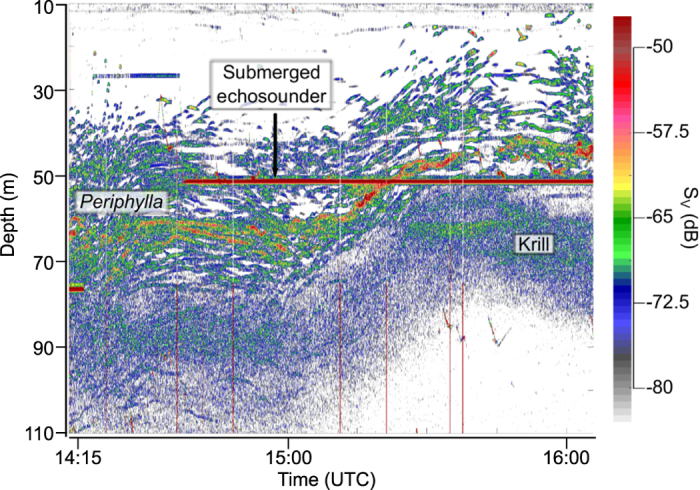
Acoustic records made from RV “Trygve Braarud”s echosounder (38 kHz) in the afternoon 12 October 2006. The echogram depicts targets ascribed to dielly migrating *Periphylla periphylla* at the upper fringe of a migrating scattering layer of krill. A submerged echosounder is seen as a horizontal line at 50 m depth during most of the registration period. Colour scale refers to backscattering strength (S_V_), with brownish-red representing the strongest echoes.

*2006:* Acoustic studies were made one afternoon using the echosounders of the RV “Trygve Braarud” and a submerged echosounder, kept at 50 m most of the registration period. The 38 kHz echosounder of the RV revealed individual targets (jellyfish; see Methods) below ~40 m, with a core of particularly strong echoes at 60–70 m. More diffuse echoes (krill) appeared below (Fig. 1). The echogram from the RV also displays the trace of the submerged echosounder. As the dielly migrating targets ascended towards the surface, the core of dense registrations passed the submerged echo sounder (Fig. 1).

**Figure 2:**
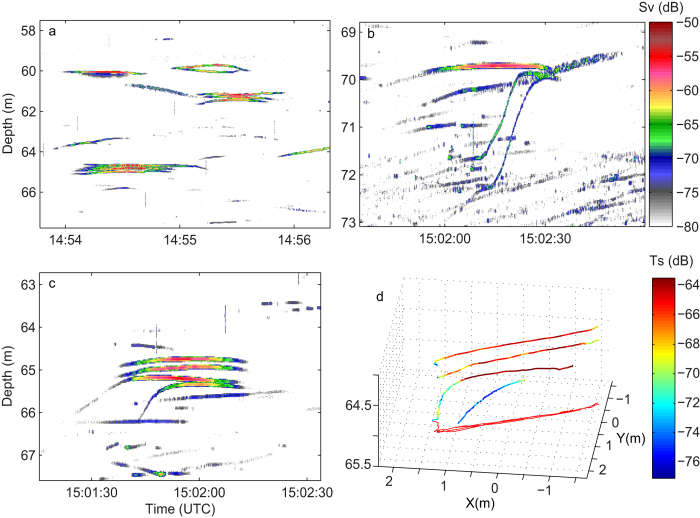
Acoustic records made from a 200 kHz echosounder, deployed at 50 m depth in Lurefjorden on 12 October 2006. Each echo trace is ascribed to *Periphylla periphylla*. **a**) 3 groups of closely spaced individuals **b**) 2 individuals ascending towards a jellyfish above, forming a group of 3 individuals **c**) Group of 2 individuals being joined by 2 more individuals, forming a group of 4 **d**) Same group as in “c”, but in 3-D, showing that individuals align just beneath each other. Colour scale for “a-c” refers to backscattering strength (S_V_); colour scale for “d” refers to individual target strength (Ts), with brownish-red representing the strongest echoes in both cases.

High resolution data from the submerged echo sounder (200 kHz) show that acoustic targets (jellyfish) in the core of the layer occurred pair-wise, or in small groups (Fig. 2). This likely explains the strong echoes from the hull mounted transducer, which has too coarse vertical resolution to distinguish between the individuals in these asssemblages, thereby giving added echo strength. On several occasions individuals could be observed as they actively joined already existing groups (Fig. 2b,c). In such cases, an individual ascended from below and positioned itself aligned beneath another one (Fig. 2d). The acoustic data suggest that one jellyfish could detect a neighbour from at least 2 m below. This vertical distance is suggested from the observations of individuals, which abruptly changed their behaviour and rapidly ascended when passing beneath another speciemen at this range (Fig. 2b). The two ascending individuals displayed in Fig. 2b swam vertically at speeds of ~17 and 18 cm s^−1^ (derived from the echogram).

Groups were detected as these acoustic targets were ascending towards the submerged echosounder in the afternoon, but not after the strong core of the vertically migrating SL had migrated past the echosounder. However, as these jellyfish approached the surface in the evening, corresponding behaviour was suggested also by the vessels’ 120 kHz echo sounder, which has a narrower beam and shorter pulse length than the 38 kHz echosounder, providing better resolution. These observations included a few examples of individuals which appeared to join groups from below and for a few meters ascended with a speed of ~10 cm sec^−1^, thereafter aligning themselves below the other members (not shown). While individuals evidently changed their behaviour to form/join a group, the groups appeared to follow the same DVM patterns as for single individuals (cf. Fig. 1).

**Figure 3:**
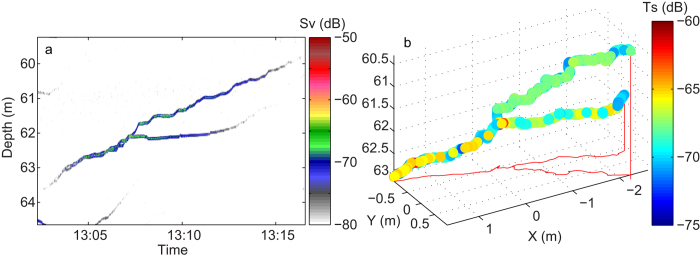
Two individuals swimming together, subsequently splitting up. Records are from a stationary, upward looking 200 kHz echosounder, floating in a buoy at 100 m depth, October 2010. Results are visualized in a normal echogram (**a**) and in 3-D based on acoustic target tracking (**b**). Colour scale for “a” refers to backscattering strength (S_V_) and colour scale for “b” refers to individual target strength (Ts), with brownish-red representing the strongest echoes in both cases.

**Figure 4:**
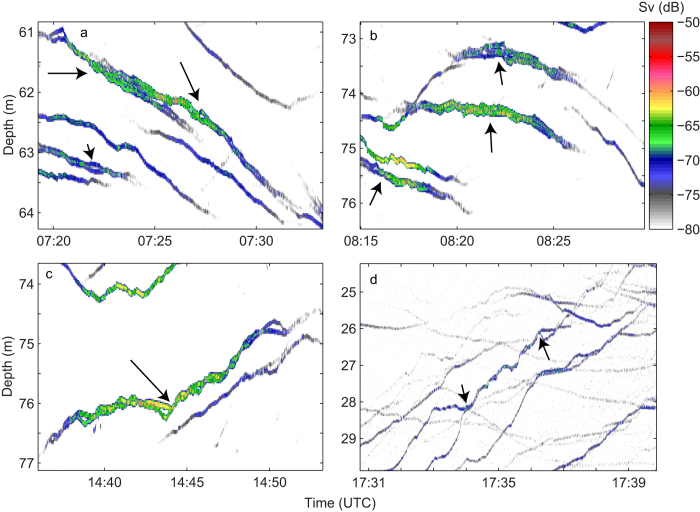
Examples of groups recorded from the stationary upward looking 200 kHz echosounder, October 2010. Examples are from descent in the morning, early day, late day and ascent in the afternoon. Arrows depict examples of group behaviour; arrows in “d” suggest the formation and splitting of a group of two individuals. Colour scale refers to backscattering strength (S_V_), with brownish-red representing the strongest echoes.

*2010.* Based on the results from 2006, jellyfish in the upper 100 m appeared to be the ones forming groups. Therefore, an upward-looking echosounder (200 kHz) was deployed in a buoy floating at 100 m depth. This depth was selected to obtain high resolution data for jellyfish with expected daytime distribution at 50–100 m. Group behaviour was repeatedly recorded during a 7 days registration period from this upward-looking echo sounder. Even with the short pulse length applied at 200 kHz (0.128 ms; vertical resolution 2,4 cm), individuals in groups could for most of the time not be resolved as the acoustic targets (jellyfish) stayed too tightly together. Groups appeared as stronger and slightly more vertically extended echo traces than individual jellyfish, but occasionally sufficient separation between individuals forming a group made it possible to detect them separately (Figs 3,4).

The acoustic data contained examples of both group formation and separation (Figs 3,4). We could not decide any typical “life span” of a group, as groups would normally drift by and not spend sufficiently long time in the acoustic beam to assess both their formation and separation. Therefore, the duration of observations would rather reflect current speed than jellyfish behaviour. The maximum duration in our data was ~20 minutes (until the group drifted out of the acoustic beam). However, also short durations were detected, with individuals joining, and separating after a few minutes (Fig. 4).

In total, 152 groups were observed during the registration period, while ~4000 echo traces of single individuals were recorded in the course of the same period. This suggests that ~10% of the jellyfish in the upper part of the water column were engaged in group behaviour, as each group normally appeared to consist of 2–3 individuals. Groups were observed during descent in the morning, daytime and ascent in the afternoon. No groups were observed at night.

### Visual observations

**Figure 5:**
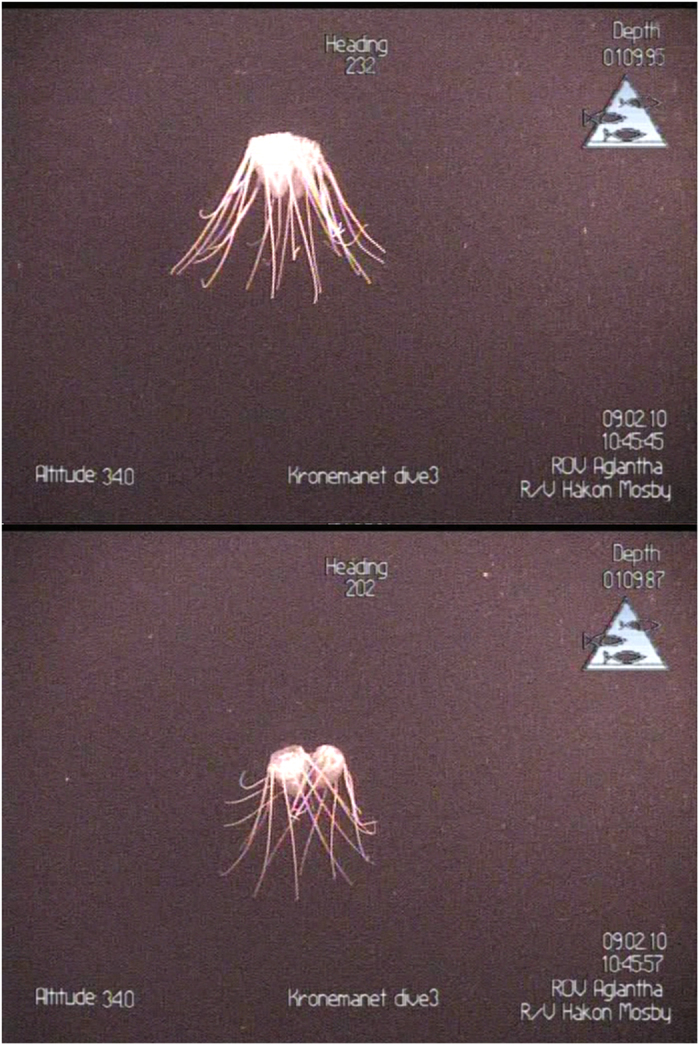
Pictures of a pair of *Periphylla periphylla* (from ROV video) taken 12 sec apart (109 m depth).

In February 2010, direct visual observations of groups were made at the surface, as well as with the ROV which documented that *P. periphylla* would form groups also at depth (Fig. 5). In total 5 pairs of individuals with their tentacles entangled were recorded with the ROV. Quantifications of size or distances were not possible, thus any other forms of group behaviour (jellyfish swimming close to each other without physical contact) could not be adequately assessed. Yet it was evident that the vast majority of the filmed jellyfish occurred as single individuals during these records in winter.

## Discussion

The current study has documented apparent social behaviour among a jellyfish. The observations of group-forming jellyfish adds to the conspicuously varied individual behaviour within this population^5^,^10^ and in jellyfish at large (see below). Tiemann *et al.* 9 made visual observations of clusters of *P. periphylla* at the surface at night, with individuals on occasions having their tentacles entangled, as also observed – but not being the focus - in the current study. Here we show that individuals team up also at depth.

The behavioural observations support that the encounters are not entanglements of chance, but active group forming. Jellyfish are basal metazoans and thought to have very limited sensory capabilities (but see^11^,^12^) and never thought to have social interactions. The findings reported here challenge our current notions about jellyfish behaviour and raise questions about the cue(s) that the jellyfish use to seek each other out at depth as well as about the adaptive values of group behaviour. In our observations of individuals joining another individual, or an already existing group, individuals joined from below in daylight or at dusk. The reaction distance would be at least ~2 m, derived from the observed behavioural changes in response to other jellyfish/groups at this range (e.g. Fig. 2). Recognizable physical disturbance from a swimming jellyfish is on the scales of cm’s^13^ and is not likely to extend several meters. The approaching *Periphylla* did not seem to follow the trail of its conspecific (Fig. 2), as seems to be the case among small plankton following chemical tracks^14^, but rather ascended abruptly when passing below upcoming partners.

Many jellyfish are bioluminecent, which is traditionally believed to be a warning to potential predators^15^,^16^. Jarms *et al.*^15^ assumed that bioluminescence as part of social interactions was unlikely in *P. periphylla*, because of its simple photoreceptors. However, the wide repertoire of bioluminescent displays of *P. periphylla*^16^ may indicate multiple functions and many other deep sea animals use bioluminescence for intraspecific communication^17^,^18^. One way of testing for intraspecific bioluminescent communication in jellyfish *in situ* might be to record their various bioluminescent signals in the laboratory, and display these to the jellyfish in the field, studing their response by means of a stationary, submerged echosounder.

Bioluminescence functions best in darkness^19^ and our registrations were in the upper part of the water column during times with surface light. Alternatively, the jellyfish might locate the shadow/silhouettes of conspecifics towards the surface. When detecting a conspecific, individuals abruptly ascended to join a jellyfish directly above (Fig. 2), the ascent speed in such cases being about 5–10 times their normal vertical swimming speed^20^.

Many marine animals with dilute populations have sophisticated adaptations for locating conspecifics, often for mating. Tiemann *et al.*^9^ speculated that *P. periphylla* migrating to the surface would increase mate encounter when moving from 3 to 2 dimensions, and that this behaviour was developed in response to low abundances in oceanic environments. However, we here show that surface encounters are not a necessity to seek out conspecifics. While mating is a plausible function, alternative explanations may apply. Also, groups forming at depths are not necessarily equivalent to those forming at the surface. Likewise, motivation for teaming up when faced with high concentrations of prey may differ from motivation outside of prey patches, or when faced with predators.

The records of group-forming jellyfish were made at the upper fringe of an acoustic scattering layer of the krill *Meganyctiphanes norvegica*, which is known as prey for *P. periphylla* in Lurefjorden^4^,^21^. Predators of various taxa may accumulate in respons to local prey aggregations (e.g.^22^). These may not be social aggregations as such but simply a response to a common cue related to e.g. prey density. However, while jellyfish may have accumulated at the top of the krill layer for favourable feeding conditions, the very close associations and coherent behaviour of the groups do not support that plain aggregation in prey patches would be the explanation for the group behaviour reported here.

Jellyfish swimming behaviour is often related to feeding strategy (e.g.^21^,^23^,^24^). As tactile predators, jellyfish rely on collisions with prey. Encounters are mediated by predator, prey^25^, or water motion^26^. *P. periphylla* swims slowly with its tentacles first, allowing the jellyfish to approach prey with a minimum amount of fluid disturbance caused by swimming^13^,^21^. Nevertheless, escapement is prevalent when foraging on agile organisms. For example, krill jump in random directions in response to contact with tentacular predators^27^. Prey escapes from one predator may therefore enhance the chance of capture by its neighbour, and *vice versa,* thus benefiting both individuals in the long run. To evaluate if instantaneous prey escapes - elicited by one predator – enhance the success of a neighbour, we formulated a probabilistic model of cooperation (Table 1). That exercise suggests that hunting in groups indeed may be profitable. However, the fact that groups are ephemeral and vary temporally and with depth may suggest that there also are significant costs involved with this behaviour.

Regardless of the adaptive significance, our data imply active social behaviour among a jellyfish that normally lives in the deep sea and that they have ways of locating and finding each other in the open water masses. Although jellyfish are simple organisms, with poorly developed sensory capabilities and neural networks, jellyfish may display well-developed behavioural repertoires^28^,^29^,^30^. Horizontal migrations guided by the position of the sun have been recorded in several species^28^,^31^, vertical migration may prevent tidal dispersal^29^ and recent studies of jellyfish using electronic tracking tags have revealed directional swimming related to current flows that may help in bloom maintenance in apparent favourable habitats^32^. Such direct tracking of jellyfish has also unveiled vertical movements that seem linked to searching the water column for prey, and that jellyfish can search the water column like fish^33^. Our results on social behavior of *P. periphylla* add to the emerging pattern that jellyfish are not simply passive drifters but rather display active and varied behavioural repertoirs of adaptive significance.

## Methods

Results in this paper are based on observations from 3 cruises to Lurefjorden. In October 2006, small groups of acoustic targets interpreted as *Periphylla periphylla* were observed during their ascent in the afternoon. During a cruise in February 2010 the primary aim was to film groups of *P. periphylla* using an ROV. During this cruise we also made abundant observations of *P. periphylla* forming groups at the surface at night (cf.^9^). In September/October 2010 we conducted more comprehensive acoustic studies to further document the group behaviour. Additional video footages from ROV were made 31 October 2011; abundant records of single individuals were made, but no groups were observed during that field effort, which is not further referred to here.

### Acoustic studies

#### October 2006

The studies were carried out during the field campaign described by^5^. Results presented here were obtained while the 22 m RV *Trygve Braarud* (University of Oslo) was freely drifting in the calm fjord waters. Acoustic records were made with the ships’ hull mounted Simrad EK500 38 kHz (11.8° opening angle) and 120 kHz (7°) echo sounders, and with a submerged Simrad EK60, 200 kHz (7°) cabled to a PC on board. The EK60 was deployed at various depths down to 150 m, with measurements both day and night. This comprised measurements above and below a relatively dense scattering layer observed by the ships’ echosounder. This layer passed the submerged echosounder at 50 m depth when ascending in the afternoon (Fig. 1). The submerged echosounder was operated with a short pulse length (0.128 ms) for the highest possible vertical resolution (2.4 cm).

#### September/October 2010

An upward-looking Simrad EK60, 200 kHz echosounder was deployed in a buoy floating at 100 m depth. This depth was selected to obtain high resolution data for jellyfish with expected daytime distribution at 50–100 m. The buoy was anchored to the bottom (near the location of the acoustic studies by^5^ and the echosounder was cabled to shore for power and transfer of data during the deployment (27 September to 3 October). The echo sounder was operated at short pulse length (128 ms) with a ping rate of 2 s^−1^. From these acoustic records we documented the formation of groups and roughly quantified the fraction of *P. periphylla* engaging in groups.

Upward-facing echosounders cabled to shore were also located at 200 m (120 kHz) and at the bottom (290 m; 38 kHz). Echograms were visually inspected without detecting apparent groups of *P. periphylla* in the deeper part of the water column covered by these two moorings.

#### Processing acoustic data

The presence of groups was assessed by visually scrutinizing echograms made using the software Sonar 5 Pro version 6.0.0^34^. This software was also applied for acoustic target tracking (TT) and for assessing target strength (TS), the latter being a proxy for size. TT groups individual echoes into tracks utilising information on the proximity of sequential echoes in determining the tracks so that a particle can be followed both horizontally and vertically as it moves through the acoustic beam^35^. Presentations of these 3-D data are based on smoothed data (linear interpolation). Echograms for the figures were made in Matlab.

#### Video and photographic records

Video records were made in February 2010. Vertical casts throughout the water column were carried out with the ROV “Aglantha”, equipped with cameras for both normal and red light, the results reported here from use of the latter.

#### Sampling

Trawling for identification of acoustic targets was done in 2006; *P. periphylla* completely dominated trawl catches by weight at 50–100 m^5^. The prevalence of *Periphylla* accords with numerous studies from Lurefjorden^3^,^4^,^36^ as well as with ROV records in this study.

## Additional Information

**How to cite this article**: Kaartvedt, S. *et al.* Social behaviour in mesopelagic jellyfish. *Sci. Rep.* **5**, 11310; doi: 10.1038/srep11310 (2015).

## Figures and Tables

**Table 1:** A simplified model of enhanced prey encounter for *Periphylla periphylla* swimming in pair and foraging on krill which is assumed to be able to escape in 2 dimensions.

The capture of prey may be regarded as a counting process with intensity λ, so *N*(t) = number of krill caught up to time t will be a Poisson counting process with parameter λt. In particular, the expected catch up to time t is λt.
The sum *N*(t) = *N*_1_(t)+*N*_2_(t) of two different counting processes *N*_1_ and *N*_2_ with intensities of λ_1_ and λ_2,_ respectively (as for example for the total number of krill caught by two different *Periphylla periphylla*) is also a Poisson process with intensity λ = λ_1_ + λ_2_.
For simplicity we assume each *P. periphylla* has equal catch intensity λ. Swimming pairwise may infer a cost (a relative reduction of ε in predator efficiency), for example hydrodynamic, with the new catch intensity λ – ελ = (1-ε)λ.
On the other hand, each jellyfish will experience an enhanced prey field as the neighbour is scaring adjacent prey. Therefore, each of the two jellyfishes will experience an extra counting process of some ‘cooperative’ intensity θλ. Hence, when going tandem, the jellyfish predation is composed of two independent Poisson processes; one within its own tentacle field with intensity (1-ε)λ and another due to scaring by its companion with intensity θλ. Each jellyfish in tandem will therefore experience a catch intensity of
(1-ε)λ + θλ = (1 – ε + θ)λ.
From this expression of the changed value of the feeding intensity of individuals swimming in tandem, it follows that cooperation will have a selective advantage if θ > ε.
This inequality is satisfied if the disadvantage of tandem swimming is minute, and the ‘passive’ catching of extra prey escaping from the neighbour will be of the order 25% of a single individual’s catch intensity. That is, half of the prey are on the neighbour-side and half of these jump out of the tentacle-cone; that is half of the half, i.e. 25%, are lost to the neighbour. But, by symmetry, they also gain 25% in the counter direction. The benefit decreases if letting krill escape in 3 dimensions and increases if increasing group size.

According to (21), the estimated daily food requirement of an individual *P. periphylla* is on the order of 1 *Meganyctiphanes norvegica*. This implies that catch rates of a prey krill can be considered as a rare event, and therefore be modeled as a Poisson process with a low intensity.
